# Second primary cancers among 109 000 cases of non-Hodgkin's lymphoma

**DOI:** 10.1038/sj.bjc.6602654

**Published:** 2005-06-21

**Authors:** P Brennan, G Scélo, K Hemminki, L Mellemkjaer, E Tracey, A Andersen, D H Brewster, E Pukkala, M L McBride, E V Kliewer, J M Tonita, A Seow, V Pompe-Kirn, C Martos, J G Jonasson, D Colin, P Boffetta

**Affiliations:** 1 International Agency for Research on Cancer, 69008 Lyon, France; 2 German Cancer Research Center, 69120 Heidelberg, Germany; 3 Department of Biosciences at Novum, Karolinska Institute, 14157 Hudinge, Sweden; 4 Institute of Cancer Epidemiology, Danish Cancer Society, 2100 Copenhagen, Denmark; 5 New South Wales Cancer Registry, 2015 Eveleigh, New South Wales, Australia; 6 Institute of Population-Based Cancer Research, 0310 Oslo, Norway; 7 Scottish Cancer Registry Information Services, NHS National Services Scotland, EH53SQ Edinburgh, Scotland; 8 Finnish Cancer Registry, Institute for Statistical and Epidemiology Cancer Research, 00170 Helsinki, Finland; 9 Cancer Control Research Programme, British Columbia Cancer Agency, V5Z4E6 Vancouver, British Columbia, Canada; 10 Epidemiology and Cancer Registry, CancerCare Manitoba, R3E0V9 Winnipeg, Canada; 11 Community Health Sciences, University of Manitoba, R3E0V9 Winnipeg, Canada; 12 Program Evaluation and Surveillance, Saskatchewan Cancer Agency, S4T7T1 Regina, Saskatchewan, Canada; 13 Center for Molecular Epidemiology, 117597 Singapore, Singapore; 14 Cancer Registry of Slovenia, Institute of Oncology, 1105 Ljubljana, Slovenia; 15 Cancer Registry of Zaragoza, Health Department of Aragon Government, 50004 Zaragoza, Spain; 16 Icelandic Cancer Registry, Icelandic Cancer Society, 125 Reykjavik, Iceland; 17 Faculty of Medicine, University of Iceland, 125 Reykjavik, Iceland

**Keywords:** non-Hodgkin's lymphoma, second primary cancers, cancer registries

## Abstract

An analysis of other primary cancers in individuals with non-Hodgkin's lymphoma (NHL) can help to elucidate this cancer aetiology. In all, 109 451 first primary NHL were included in a pooled analysis of 13 cancer registries. The observed numbers of second cancers were compared to the expected numbers derived from the age-, sex-, calendar period- and registry-specific incidence rates. We also calculated the standardised incidence ratios for NHL as a second primary after other cancers. There was a 47% (95% confidence interval 43–51%) overall increase in the risk of a primary cancer after NHL. A strongly significant (*P*<0.001) increase was observed for cancers of the lip, tongue, oropharynx^*^, stomach, small intestine, colon^*^, liver, nasal cavity^*^, lung, soft tissues^*^, skin melanoma^*^, nonmelanoma skin^*^, bladder^*^, kidney^*^, thyroid^*^, Hodgkin's lymphoma^*^, lymphoid leukaemia^*^ and myeloid leukaemia. Non-Hodgkin's lymphoma as a second primary was increased after cancers marked with an asterisk. Patterns of risk indicate a treatment effect for lung, bladder, stomach, Hodgkin's lymphoma and myeloid leukaemia. Common risk factors may be involved for cancers of the lung, bladder, nasal cavity and for soft tissues, such as pesticides. Bidirectional effects for several cancer sites of potential viral origin argue strongly for a role for immune suppression in NHL.

The incidence of non-Hodgkin's lymphoma (NHL) has increased in most parts of the world (Bray *et al*, 2001). A comparison of cancer registry information between 1982 and 1997 (Parkin *et al*, 1992, 2002) indicates that this increase is occurring at an average annual rate of 4–5% each year, implying a doubling of NHL incidence every 20 years. This upward trend has been observed in all geographical regions covered by cancer registration, and is not restricted to any particular age group or sex, or to predominantly rural or urban areas. The reason for the increase has attracted much speculation although there is no clear explanation for it.

It has long been recognised that clusters of second primary cancers provide a unique clue to the understanding of cancer aetiology and mechanisms (Hanlon, 1931). If detection and other biases can be excluded, then the increased risk of an individual developing more than one primary cancer may be attributed to either (i) common risk factors between the cancers including environmental exposures and genetic factors, or (ii) effects of treatment, particularly chemo- and radiotherapy for the first primaries (Boice *et al*, 1985). It is often possible to distinguish between these two explanations. Cancers that share a common aetiology are likely to be increased after each other, whereas an increased incidence of treatment-related second primary cancers is often unidirectional. Furthermore, an increase in treatment-related cancers usually only becomes apparent years after the first primary cancer. To help elucidate the potential causes of NHL and the recent increasing incidence, we have therefore studied the occurrence of second primary cancers in over 109 000 patients with NHL from 13 cancer registries.

## MATERIALS AND METHODS

In order to conduct a systematic analysis of second primary cancers, an international multicentre study has been initiated incorporating large cancer registries which have been in operation for at least 25 years. In all, 19 cancer registries that have consistently reported their cancer incidence figures in Cancer Incidence in Five Continents (Parkin *et al*, 2002) were invited to participate. A similar analysis is underway in the US SEER cancer registries and they are therefore not included in this analysis. Consistent publication in consecutive versions in Cancer in Five Continents was taken as a proxy measure of quality of the cancer registry, including high levels of morphological verification and low levels of cancers identified only through death certificates. Of an initial group of 19 contacted registries, 15 confirmed that the project was feasible and provided all necessary data. Two registries were subsequently excluded because of discrepancies in the observed rates of second primaries, leaving 13 registries in the current analysis. These registries are British Columbia, Manitoba and Saskatchewan (Canada), New South Wales (Australia), Singapore, Norway, Denmark, Sweden, Finland, Iceland, Scotland, Slovenia and Zaragoza (Spain). Some individual analyses with partial overlap with the current data set have previously been reported (Storm and Prener, 1985; Adami *et al*, 1995; Brennan *et al*, 2000; Dong and Hemminki, 2001; McKenna *et al*, 2003).

Anonymised data were provided from each cancer registry on all initial primary cancers, including age and sex of the subject, diagnosis and date of the first primary, follow-up for mortality and date and diagnosis of the second primary, if any. Information was also obtained from each cancer registry on the set of rules used for defining a second primary cancer. As these differ between cancer registries, and also over time, the International Association of Cancer Registries (IACR)/International Agency for Research on Cancer (IARC) rules on second primary cancers were adopted as a common set of rules (Muir and Percy, 1991). This was possible as all participating cancer registries currently use the IACR/IARC rules, or a local set of more extensive or detailed rules.

All cases of first primary NHL were followed up for second primary cancer from the date of NHL diagnosis (1943–2000), to the date of second primary cancer (1943–2000), date of death, date of migration or end of follow-up (1992–2000). To assess any possible excess of second primary neoplasms after NHL, we compared the observed number of neoplasms to the expected number derived from the age-, sex- and calendar period-specific cancer incidence rates of first primary cancers in each of the cancer registries. Standardised incidence ratios (SIRs) adjusted for age, year, sex and registry were calculated using indirect standardisation methods. Exact confidence intervals (CI) around the SIR were calculated assuming a Poisson distribution for the observed number of neoplasms. For those cancer sites that were significantly increased after NHL (*P*<0.001), detailed SIRs were calculated after stratifying for age, follow-up period and calendar period. Finally, we have calculated the SIRs for NHL as a second primary after other cancer sites as a first primary.

## RESULTS

The population included 109 451 NHL first primary cases and 7427 NHL cases as a second primary cancer (Table 1). Among the first primary NHL cases, 37% provided less than 1 year of follow-up, whereas 13% provided at least 10 years of follow-up. Among the 13 registries, 19% of the first primary NHL cases came from Sweden, with substantial contributions also from Denmark (15%), New South Wales (14%), Norway (13%), Scotland (10%), Finland (10%) and British Columbia (9%).

Among the 109 451 NHL first primary cases, there was a 47% overall increase in the risk of a second primary cancer (SIR=1.47, 95% CI 1.43–1.51) (Table 2). This relative risk was higher with increasing time of follow-up, being 1.37 (95% CI 1.32–1.43) in the 1–4-year follow-up period, and 1.67 (95% CI 1.59–1.76) after 10 years or more (test for trend *P*<0.05).

A strongly significant (*P*<0.001) increase was observed for 18 separate cancers: lip, tongue, oropharynx, stomach, small intestine, colon, liver, nose and nasal cavity, lung, soft tissue, skin melanoma and nonmelanoma skin cancer, bladder, kidney, thyroid, Hodgkin's lymphoma, lymphoid leukaemia and myeloid leukaemia. Among the 960 nonmelanoma skin cancers that occurred after the first NHL, 36.9% were basal cell cancers and 1.5% were Kaposi's sarcomas. Further attention is restricted to these 18 cancer sites.

When second cancer risk was stratified by gender, there was some evidence of a higher risk among men than women (SIR=1.50 *vs* 1.43, *P* for heterogeneity=0.06), which was mainly due to skin melanoma (SIR=2.20 *vs* 1.52, *P*<0.01), skin nonmelanoma (SIR=3.77 *vs* 2.61, *P*<0.01) and multiple myeloma (SIR=1.30 *vs* 0.69, *P*=0.02). Conversely, women had a higher risk of lip (SIR=4.07 *vs* 1.58, *P*<0.01), stomach (SIR=1.65 *vs* 1.17, *P*<0.01), colon (SIR=1.47 *vs* 1.13, *P*<0.01) and lung cancers (SIR=1.72 *vs* 1.39, *P*<0.01).

We investigated whether any excess risk was constant over time or whether it increased with follow-up, the latter being more typical of a treatment effect. After excluding the first 12 months of follow-up, a significant (*P*<0.05) increasing trend was observed for only stomach, lung, bladder cancer and Hodgkin's lymphoma, though for all four sites an increase in risk was also seen in the initial 1–4-year period, indicating that either the trend was not solely a treatment effect, or alternatively that latent period for the treatment effect could be as little as 4 years. An increased risk was also observed with myeloid leukaemia, which was most prominent between 5 and 9 years after NHL onset.

Six of the cancer sites showed a decreasing risk with increasing age of NHL onset, these being stomach, lung, bladder, Hodgkin's lymphoma, lymphoid leukaemia and myeloid leukaemia (Table 3). None of the cancer sites showed an increasing risk with increasing age of onset of NHL. Regarding year of onset, few of the cancer sites showed any increasing or decreasing trends with risk (Table 4); the exceptions were lip and kidney, both of which showed moderately higher relative risks after NHL diagnosed in the later follow-up periods.

Finally, the risk of NHL after all other cancer sites was also assessed (Table 5). Restricting attention to the 18 cancers that were increased after NHL, 11 of these also showed increases, including oropharynx, colon, nose and nasal cavity, soft tissue sarcoma, skin melanoma and nonmelanoma skin cancer, bladder, kidney, thyroid, Hodgkin's lymphoma and lymphoid leukaemia.

## DISCUSSION

Our results show a 47% (95% CI 43–51%) overall increase in the risk of primary cancer after NHL. The different patterns of risk for the 18 cancers with an overall increased incidence are summarised in Table 6. This analysis of second primary cancers after NHL suffers from several limitations. Non-Hodgkin's lymphoma is really a cluster of separate cancers and the lack of information on subtypes of NHL removes the possibility of identifying more specific relationships. We could not attempt any extensive analysis by subtype due to the differing amount of subtype information in individual registries, as well as the likely differing quality of subtype information both between registries and over time. We also have not included in this analysis any information on treatment, because of the very limited and heterogeneous amount of information available in all of the cancer registries. Particular care was taken to standardise cancer site definitions between registries and over time, although some heterogeneity among results in the 13 registries may have occurred due to differences in treatment and exposure, as well as specific cancer registry characteristics. The latter source of heterogeneity was minimised by ensuring a common protocol across the registries for reporting second primaries, detailed comparison of results to identify discrepancies and the dropping of two registries due to apparent under-reporting in one and over-reporting in the other that could not be easily explained.

### Treatment effects

Five of the cancer sites (lung, bladder, stomach, Hodgkin's lymphoma and myeloid leukaemia) showed patterns of risk consistent with a treatment effect, including an increase in risk with time since NHL diagnosis and a greater risk with young age of onset, when treatments are likely to be more intensive. Treatment regimes for NHL typically involve chemotherapy for middle- and high-grade subtypes, and localised radiotherapy or no active treatment for low-grade subtypes (Travis *et al*, 1991). The increase in stomach cancer risk was mainly observed at least 10 years after initial NHL diagnosis and may be due to initial radiotherapy treatment, as gamma radiation has been shown to increase the risk of stomach cancer (IARC Monographs Vol 75, 2000). The increasing risk of lung cancer after NHL may be also due to radiotherapy, although radiogenic lung tumours typically require a long latent period and the effect here was observed in the first 4 years after NHL diagnosis. This suggests that a common risk factor may also be present. The increasing risk of bladder cancer after NHL is likely to be explained by treatment with cyclophosphamide (IARC Monographs Suppl 7, 1987). Of interest was a small but significant increase in risk of NHL after bladder cancer, which could also indicate some joint exposures for the two cancers. Although smoking is an attractive putative common risk factor between NHL, bladder and lung cancer, the evidence for a role of smoking in NHL has been very limited until now, with only few studies suggesting that smoking may be associated with follicular NHL (Herrinton and Friedman, 1998; Parker *et al*, 2000; Morton *et al*, 2003; Stagnaro *et al*, 2004).

The excess of Hodgkin's lymphoma after NHL increased with time and was more apparent among NHL cases with a younger age at onset. When the reverse relationship was studied, a strong but relatively constant risk of NHL was observed after Hodgkin's lymphoma. The strong increase in the immediate post-diagnostic period, and the inverse relationship of NHL after Hodgkin's lymphoma would argue strongly for common exposures. While the aetiology of Hodgkin's lymphoma is not totally clear, altered immune function and specifically late exposure to common viruses and Epstein–Barr virus (EBV) are thought to play a role, and may also be involved in NHL. It should also be noted that, given the complexities of lymphoma diagnosis, some level of misclassification between Hodgkin's lymphoma and NHL is inevitable (Travis *et al*, 1992). Regarding myeloid leukaemia, the increase was mainly restricted to the 5–9-year period after NHL diagnosis, and was strongly related to young age at NHL onset. Previous studies have shown that myeloid leukaemia can be increased after NHL due to specific chemotherapy regimes including the multi-drug protocol MOPP (nitrogen mustard, vincristine, procarbazine and prednisone).

### Ultraviolet (UV) light or altered immunity

The relationship with skin cancer was bi-directional, and similar for both melanoma and nonmelanoma, which argues strongly against detection bias as an explanation for the increase. Instead, the joint association is likely to be due to a common risk factor or shared mechanism that is stronger during the initial period of diagnosis. The role of UV light has previously been put forward as explaining this joint association (Cartwright *et al*, 1994; McMichael and Giles, 1996). However, recent evidence fails to support such an association (Hughes *et al*, 2004; Smedby *et al*, 2005). The joint association with skin cancer may be due instead to a common mechanism such as general immune suppression, possibly modulated by UV exposure in the case of skin cancers, but by other exposures for NHL. It is of interest that skin cancers are strongly increased among subjects who experience extreme immune suppression, indicating an infectious aetiology for some of these cancers (Hemminki *et al*, 2003). Human papillomavirus (HPV) types 5/8 have also been identified in nonmelanoma skin cancers among transplant recipients (IARC Monographs Vol 64, 1995).

The increase in cancer of the oropharynx after NHL was relatively constant over time, and was mirrored by a similar increase in NHL after an initial diagnosis of oropharynx cancer. The excess risk may be linked to the viral aetiology of oropharyngeal cancer via moderate immune suppression. A substantial proportion of oropharyngeal cancers are thought to be related to HPV infection, in particular HPV 16 (IARC Monographs Vol 64, 1995). Similarly, the excess of liver cancer may be related to mild immunosuppression or even more directly by infection with hepatitis C, which has been recently shown to be involved with NHL (Negri *et al*, 2004). If immune suppression is the common mechanism involved in the excess risk of skin, oropharyngeal and hepatic cancers, then chemotherapy might be partly responsible for that, as many forms of chemotherapy are also immunosuppressive.

### Other common risk factors

Other cancer sites with strong bi-directional effects included nasal cavity cancer, soft tissue sarcoma and thyroid cancer. Regarding soft tissue sarcoma, its aetiology is little understood although an increase in risk has been reported among workers exposed to phenoxy acid herbicides (Kogevinas *et al*, 1997), which has also been linked with NHL. The epidemiology of thyroid cancer is poorly understood, with ionising radiation, particularly at a young age, being the one consistently observed risk factor. However, it is unlikely that ionising radiation could explain the joint association between these cancers given that it does not appear to increase the risk of NHL. It is however possible that both cancers may share a common genetic pathway given the well-known increased risk of NHL among ataxia-telangiectasia (AT) homozygotes, and a possible increased risk of thyroid cancer among relatives of AT patients (Geoffroy-Perez *et al*, 2001), although this observation was based on one case only. Investigation of DNA repair genes in NHL, in particular double-strand break repair, would therefore appear to be of interest.

### Detection and misclassification bias

Given the bi-directional association between NHL and kidney cancer, common exposures for the two cancers may be suspected. However, the increase in kidney cancer after NHL was strongly concentrated on the first 12 months after diagnosis, indicating a possible detection bias. A similar increased risk of kidney cancer after NHL that was largely restricted to the initial follow-up period was also observed among 29 153 SEER NHL patients that was thought to be probably explained by detection bias (Travis *et al*, 1991). The excess risk of small intestine cancer after NHL was also concentrated in the first 12 months after NHL diagnosis, again arguing for detection bias. Similarly, the increase in lymphoid leukaemia after NHL is likely to be partially explained by misclassification between these two sites as well as some cases of NHL developing into lymphoid leukaemias.

Extra-nodal lymphomas may also be classified according to the primary site resulting in spurious associations. It has been estimated that up to 33% of NHL are extra-nodal, with the most common sites being stomach, skin, small intestine and oropharynx (Newton *et al*, 1997). Primary cancers at all of these sites were increased after NHL in the current analysis. Given that nodal and extra-nodal NHL have been classified separately only with more recent classification schemes, we have been able to ascertain nodular status for 24 018 of the 109 451 NHL patients (registries that utilised ICDO-1 or ICDO-2 codes, wholly or partly included British Columbia, Saskatchewan, Scotland, Spain and New South Wales): 18 812 were nodular, 1782 originated from other lymphatic tissues (such as tonsils, spleen, thymus and Waldeyer's ring) and 3424 were extra-lymphatic. The overall increased risk of a second primary cancer was 1.34 (95% CI 1.26–1.42) after nodular NHL, 1.45 (95% CI 1.21–1.72) after other lymphatic tissue NHL and 1.37 (95% CI 1.20–1.72) after extra-nodular NHL. Of the 18 sites, the 10 that increased after all NHL were still significantly increased after nodular NHL (all except lip, stomach, small intestine, colon, liver, nasal cavity, bladder and myeloid leukaemia). The absence of an increased risk for these eight sites is likely to be due to the much reduced power resulting from the smaller sample size. However, it is of interest to note that increased risks after extra-lymphatic NHL were observed for both colon (SIR=1.59, 95% CI 1.02–2.37) and stomach (SIR=1.89, 95% CI 0.91–3.48), and the misclassification with cancer from the corresponding organ is likely to explain the overall increased risk of colon and stomach cancer after NHL.

In conclusion, this analysis of second primary cancers after NHL provided evidence for a treatment-related effect for cancers of the bladder, lung, stomach, myeloid leukaemia and Hodgkin's lymphoma. Nontreatment-related associations were observed for melanoma and nonmelanoma skin cancer, for which immune suppression as a common underlying mechanism is a strong candidate. Exposure to pesticides deserves further attention in individual level studies, as suggested by the bi-directional association with soft tissue sarcomas.

## Figures and Tables

**Table 1:** Distribution of NHL patients as first and second cancer by sex, age, follow-up, and calendar period

	**No. of first primary NHL (%)**		**No. of second primary NHL^a^ (%)**	
*Sex*				
Women	49 808	(45.5)	3592	(48.4)
Men	59 643	(54.5)	3835	(51.2)

*Age at NHL diagnosis*				
Less than 56 years old	34 881	(31.9)	761	(10.2)
56–65 years old	23 514	(21.5)	1221	(16.4)
66–74 years old	26 368	(24.1)	2133	(28.7)
At least 75 years old	24 688	(22.6)	3312	(44.6)

*Calendar period at NHL as a first cancer*				
Before 1975	17 976	(16.4)	445	(6.0)
1975–1983	24 553	(22.4)	1133	(15.3)
1984–1990	28 828	(26.3)	2141	(28.8)
1991 or after	38 094	(34.8)	3708	(49.9)

*Follow-up duration*				
Less than 1 year	40 219	(36.7)	1192	(16.0)
1–4 years	37 273	(34.1)	2445	(32.9)
5–9 years	17 786	(16.3)	1745	(23.5)
At least 10 years	14 173	(12.9)	2045	(27.5)

*Registry (follow-up period)*				
Australia, New South Wales (1972–1997)	15 913	(14.5)	877	(11.8)
Canada, British Colombia (1970–1998)	9474	(8.7)	628	(8.5)
Canada, Manitoba (1970–1998)	3681	(3.4)	425	(5.7)
Canada, Saskatchewan (1967–1998)	2301	(2.1)	205	(2.8)
Denmark (1943–1997)	16 316	(14.9)	1282	(17.3)
Finland (1953–1998)	10 610	(9.7)	574	(7.7)
Iceland (1955–2000)	551	(0.5)	43	(0.6)
Norway (1953–1999)	14 320	(13.1)	950	(12.8)
Singapore, Chinese (1968–1992)	1822	(1.7)	33	(0.4)
Slovenia (1961–1998)	1603	(1.5)	73	(1.0)
Spain, Zaragoza (1978–1998)	1322	(1.2)	46	(0.6)
Sweden (1961–1998)	20 865	(19.1)	1608	(21.7)
UK, Scotland (1975–1996)	10 673	(9.8)	683	(9.2)

Total	109 451		7427	

NHL, non-Hodgkin's lymphoma.

a Excluding those following a NHL.

**Table 2:** Standardised incidence ratios of second primary cancers following NHL by follow-up period

	**Less than 1 year**			**1–4 years**			**5–9 years**			**At least 10 years**				**Overall**		
**Cancer sites (ICD 9th revision)**	**Obs.**	**SIR**	**95% CI**	**Obs.**	**SIR**	**95% CI**	**Obs.**	**SIR**	**95% CI**	**Obs.**	**SIR**	**95% CI**	***P* for trend^a^**	**Obs.**	**SIR**	**95% CI**
**All malignant (140–208)**	**1123**	**1.36**	**1.28–1.44**	**2440**	**1.37**	**1.32–1.43**	**1646**	**1.55**	**1.48–1.63**	**1464**	**1.67**	**1.59–1.76**	**<0.01**	**6673**	**1.47**	**1.43–1.51**
**Lip (140)**	**10**	**1.59**	**0.76–2.92**	**22**	**1.70**	**1.06–2.57**	**23**	**3.17**	**2.01–4.75**	**8**	**1.42**	**0.61–2.80**	**0.86**	**63**	**1.96**	**1.51–2.51**
**Tongue (141)**	**7**	**2.38**	**0.96–4.90**	**20**	**3.12**	**1.91–4.82**	**6**	**1.61**	**0.59–3.50**	**10**	**3.41**	**1.64–6.28**	**0.93**	**43**	**2.69**	**1.94–3.62**
**Salivary gland (142)**	**3**	**1.77**	**0.37–5.18**	**9**	**2.47**	**1.13–4.68**	**4**	**1.87**	**0.51–4.79**	**3**	**1.70**	**0.35–4.98**	**0.54**	**19**	**2.06**	**1.24–3.21**
Mouth (143–145)	4	**0.94**	0.26–2.41	17	**1.83**	1.07–2.93	7	**1.28**	0.51–2.63	6	**1.36**	0.50–2.96	*0.45*	34	**1.45**	1.00–2.03
**Oropharynx (146)**	**6**	**3.12**	**1.14–6.79**	**8**	**1.86**	**0.80–3.67**	**6**	**2.35**	**0.86–5.11**	**4**	**1.97**	**0.54–5.03**	**0.86**	**24**	**2.22**	**1.42–3.30**
Nasopharynx (147)	0	**0.00**	0.00–3.26	5	**2.09**	0.68–4.87	0	**0.00**	0.00–2.78	5	**5.08**	1.65–11.8	*0.23*	10	**1.71**	0.82–3.15
Hypopharynx (148)	0	**0.00**	0.00–2.24	5	**1.41**	0.46–3.29	1	**0.49**	0.01–2.72	4	**2.54**	0.69–6.52	*0.49*	10	**1.13**	0.54–2.09
Pharynx unspecified (149)	0	**0.00**	0.00–10.6	1	**1.34**	0.03–7.45	0	**0.00**	0.00–8.06	2	**6.69**	0.81–24.2	*0.16*	3	**1.62**	0.33–4.73
**Oesophagus (150)**	**9**	**0.80**	**0.37–1.52**	**33**	**1.42**	**0.98–2.00**	**20**	**1.47**	**0.90–2.27**	**21**	**1.98**	**1.23–3.03**	**0.27**	**83**	**1.41**	**1.13–1.75**
**Stomach (151)**	**52**	**1.21**	**0.90–1.58**	**107**	**1.27**	**1.04–1.53**	**54**	**1.13**	**0.85–1.48**	**72**	**1.91**	**1.49–2.41**	**0.02**	**285**	**1.34**	**1.19–1.50**
**Small intestine (152)**	**8**	**3.36**	**1.45–6.62**	**15**	**2.90**	**1.62–4.79**	**5**	**1.62**	**0.53–3.78**	**5**	**1.90**	**0.62–4.43**	**0.30**	**33**	**2.49**	**1.71–3.49**
**Colon (153)**	**67**	**0.97**	**0.75–1.23**	**189**	**1.26**	**1.09–1.46**	**126**	**1.39**	**1.16–1.66**	**114**	**1.51**	**1.25–1.82**	**0.12**	**496**	**1.29**	**1.18–1.41**
Rectum (154)	31	**0.74**	0.50–1.05	99	**1.10**	0.89–1.34	53	**0.99**	0.74–1.29	69	**1.55**	1.20–1.96	*0.05*	252	**1.09**	0.96–1.24
**Liver (155) (−1552)**	**17**	**2.15**	**1.25–3.44**	**21**	**1.24**	**0.77–1.90**	**13**	**1.29**	**0.69–2.21**	**16**	**1.89**	**1.08–3.07**	**0.23**	**67**	**1.55**	**1.20–1.96**
Gall bladder, bile ducts (156)	12	**1.34**	0.69–2.34	16	**0.85**	0.48–1.38	17	**1.50**	0.87–2.40	15	**1.57**	0.88–2.58	*0.07*	60	**1.23**	0.94–1.58
Pancreas (157)	23	**0.89**	0.56–1.33	51	**0.93**	0.69–1.22	36	**1.11**	0.78–1.54	40	**1.47**	1.05–2.00	*0.03*	150	**1.07**	0.91–1.25
Peritoneum (158)	3	**3.07**	0.63–8.98	2	**0.96**	0.12–3.48	1	**0.81**	0.02–4.49	0	**0.00**	0.00–3.58	*0.36*	6	**1.13**	0.41–2.45
**Nose and nasal cavity (160)**	**5**	**3.27**	**1.06–7.64**	**8**	**2.47**	**1.07–4.86**	**4**	**2.12**	**0.58–5.42**	**3**	**1.90**	**0.39–5.55**	**0.68**	**20**	**2.43**	**1.48–3.75**
Larynx (161)	3	**0.39**	0.08–1.14	18	**1.09**	0.65–1.73	13	**1.38**	0.73–2.36	15	**2.10**	1.17–3.46	*0.07*	49	**1.20**	0.89–1.59
**Lung (162)**	**137**	**1.26**	**1.06–1.49**	**300**	**1.30**	**1.16–1.46**	**230**	**1.72**	**1.50–1.96**	**187**	**1.78**	**1.53–2.05**	**<0.01**	**854**	**1.48**	**1.38–1.58**
**Bone (170)**	**2**	**1.99**	**0.24–7.17**	**5**	**2.39**	**0.78–5.58**	**3**	**2.50**	**0.52–7.31**	**4**	**4.21**	**1.15–10.8**	**0.42**	**14**	**2.67**	**1.46–4.48**
**Soft tissue sarcoma (171)**	**7**	**1.92**	**0.77–3.95**	**14**	**1.77**	**0.97–2.97**	**15**	**3.20**	**1.79–5.28**	**13**	**3.40**	**1.81–5.82**	**0.07**	**49**	**2.44**	**1.81–3.23**
**Melanoma of skin (172)**	**48**	**2.09**	**1.54–2.77**	**113**	**2.15**	**1.77–2.58**	**60**	**1.86**	**1.42–2.40**	**37**	**1.38**	**0.97–1.90**	**0.02**	**258**	**1.92**	**1.69–2.16**
**Other neoplasm of skin (173)**	**120**	**2.27**	**1.88–2.71**	**402**	**3.56**	**3.22–3.92**	**274**	**3.99**	**3.53–4.49**	**164**	**2.82**	**2.41–3.29**	**0.05**	**960**	**3.28**	**3.07–3.49**
Female breast (174)	61	**0.81**	0.62–1.04	168	**0.97**	0.83–1.13	102	**0.96**	0.78–1.17	99	**1.14**	0.92–1.38	*0.27*	430	**0.97**	0.88–1.07
Male breast (175)	2	**2.55**	0.31–9.22	2	**1.20**	0.14–4.33	1	**1.01**	0.03–5.64	2	**2.52**	0.31–9.11	*0.48*	7	**1.65**	0.66–3.41
Cervix uteri (180)	8	**0.91**	0.39–1.79	21	**1.10**	0.68–1.69	12	**1.08**	0.56–1.89	10	**1.14**	0.55–2.10	*0.94*	51	**1.07**	0.80–1.41
Placenta (181)	0	**0.00**	0.00–311	0	**0.00**	0.00–136	0	**0.00**	0.00–224	0	**0.00**	0.00–292	*NA*	0	**0.00**	0.00–54.2
Corpus uteri (182)	12	**0.72**	0.37–1.26	33	**0.87**	0.60–1.23	22	**0.96**	0.60–1.45	14	**0.73**	0.40–1.22	*0.66*	81	**0.84**	0.67–1.04
Ovary (183)	14	**0.96**	0.53–1.61	28	**0.86**	0.57–1.24	22	**1.11**	0.70–1.69	23	**1.40**	0.89–2.11	*0.07*	87	**1.04**	0.84–1.29
Other female genital (179,184)	4	**1.02**	0.28–2.60	6	**0.70**	0.26–1.52	11	**2.10**	1.05–3.76	7	**1.58**	0.64–3.27	*0.10*	28	**1.26**	0.84–1.83
Prostate (185)	134	**1.14**	0.96–1.36	243	**0.96**	0.84–1.09	161	**1.05**	0.89–1.22	169	**1.29**	1.10–1.50	<*0.01*	707	**1.08**	1.00–1.16
**Testis (186)**	**7**	**4.25**	**1.71–8.75**	**4**	**1.09**	**0.30–2.78**	**5**	**2.25**	**0.73–5.25**	**3**	**1.40**	**0.29–4.08**	**0.66**	**19**	**1.96**	**1.18–3.06**
Other male genital (187)	0	**0.00**	0.00–2.64	5	**1.70**	0.55–3.98	4	**2.36**	0.64–6.05	4	**2.77**	0.75–7.09	*0.46*	13	**1.74**	0.93–2.98
**Bladder (188,189.3,189.4)**	**52**	**1.24**	**0.93–1.63**	**108**	**1.21**	**0.99–1.46**	**85**	**1.61**	**1.28–1.98**	**98**	**2.21**	**1.80–2.70**	**<0.01**	**343**	**1.50**	**1.35–1.67**
**Kidney (189) (−189.3, 189.4)**	**114**	**5.13**	**4.23–6.16**	**52**	**1.08**	**0.80–1.41**	**44**	**1.54**	**1.12–2.07**	**29**	**1.21**	**0.81–1.74**	**0.43**	**239**	**1.94**	**1.70–2.21**
**Eye (190)**	**1**	**0.52**	**0.01–2.92**	**9**	**2.19**	**1.00–4.16**	**6**	**2.50**	**0.92–5.44**	**2**	**1.01**	**0.12–3.63**	**0.41**	**18**	**1.73**	**1.03–2.73**
**Brain, nervous system (191–192)**	**13**	**1.29**	**0.69–2.21**	**23**	**1.02**	**0.65–1.54**	**23**	**1.75**	**1.11–2.62**	**17**	**1.59**	**0.93–2.55**	**0.12**	**76**	**1.35**	**1.06–1.69**
**Thyroid gland (193)**	**13**	**2.95**	**1.57–5.05**	**16**	**1.64**	**0.94–2.66**	**12**	**2.07**	**1.07–3.61**	**15**	**3.13**	**1.75–5.16**	**0.07**	**56**	**2.26**	**1.71–2.94**
Other endocrine gland (194, 164.0)	4	**5.68**	1.55–14.5	3	**1.97**	0.41–5.77	1	**1.12**	0.03–6.22	0	**0.00**	0.00–5.12	*0.22*	8	**2.08**	0.90–4.10
**Hodgkin's disease (201)**	**10**	**3.93**	**1.88–7.23**	**19**	**3.54**	**2.13–5.53**	**21**	**6.87**	**4.25–10.5**	**19**	**7.69**	**4.63–12.0**	**0.01**	**69**	**5.14**	**4.00–6.50**
Multiple myeloma (203)	15	**1.28**	0.72–2.11	22	**0.87**	0.54–1.31	15	**0.99**	0.55–1.63	16	**1.25**	0.72–2.03	*0.27*	68	**1.05**	0.81–1.33
**Lymphoid leukaemia (204)**	**18**	**1.99**	**1.18–3.15**	**52**	**2.71**	**2.02–3.55**	**36**	**3.11**	**2.18–4.30**	**25**	**2.59**	**1.68–3.83**	**0.98**	**131**	**2.65**	**2.21–3.14**
**Myeloid leukaemia (205)**	**9**	**1.45**	**0.66–2.75**	**21**	**1.60**	**0.99–2.44**	**15**	**1.93**	**1.08–3.18**	**6**	**0.94**	**0.34–2.04**	**0.38**	**51**	**1.52**	**1.13–2.00**
**Other leukaemia (206–208)**	**16**	**3.54**	**2.02–5.75**	**32**	**3.27**	**2.24–4.61**	**19**	**3.28**	**1.99–5.12**	**16**	**3.24**	**1.85–5.26**	**0.98**	**83**	**3.31**	**2.64–4.11**
**Other malignant than defined**	**42**	**1.09**	**0.79–1.48**	**93**	**1.12**	**0.90–1.37**	**58**	**1.15**	**0.87–1.48**	**73**	**1.75**	**1.37–2.20**	**0.01**	**266**	**1.24**	**1.10–1.40**

NHL, non-Hodgkin's lymphoma.

a Excluding the category ‘Less than 1 year’.

NA, not applicable.

**Table 3:** Standardised incidence ratios of selected second primary cancers after NHL by age at NHL

	**Less than 56 years old**			**56–65 years old**			**66–74 years old**			**At least 75 years old**			
**Cancer sites (ICD 9th rev.)**	**Obs.**	**SIR**	**95% CI**	**Obs.**	**SIR**	**95% CI**	**Obs.**	**SIR**	**95% CI**	**Obs.**	**SIR**	**95% CI**	***P* for trend**
Lip (140)	8	**1.28**	0.55–2.51	18	**2.01**	1.19–3.18	26	**2.52**	1.64–3.70	11	**1.66**	0.83–2.97	*0.45*
Tongue (141)	16	**3.67**	2.10–5.96	14	**2.93**	1.60–4.92	8	**1.80**	0.78–3.55	5	**2.06**	0.67–4.80	*0.10*
Oropharynx (146)	7	**1.91**	0.77–3.94	7	**2.00**	0.80–4.13	8	**3.09**	1.33–6.08	2	**1.88**	0.23–6.80	*0.58*
Stomach (151)	59	**1.97**	1.50–2.54	81	**1.53**	1.21–1.90	85	**1.15**	0.92–1.42	60	**1.07**	0.82–1.38	<*0.01*
Small intestine (152)	10	**3.57**	1.71–6.56	8	**2.17**	0.94–4.28	10	**2.38**	1.14–4.38	5	**1.94**	0.63–4.52	*0.29*
Colon (153)	90	**1.56**	1.25–1.91	125	**1.27**	1.05–1.51	159	**1.20**	1.02–1.40	122	**1.27**	1.06–1.52	*0.18*
Liver (155) (−155.2)	16	**2.21**	1.26–3.59	15	**1.27**	0.71–2.09	21	**1.41**	0.87–2.16	15	**1.60**	0.89–2.64	*0.48*
Nose and nasal cavity (160)	6	**3.30**	1.21–7.18	6	**2.56**	0.94–5.58	4	**1.57**	0.43–4.02	4	**2.61**	0.71–6.68	*0.49*
Lung (162)	231	**2.18**	1.91–2.48	288	**1.59**	1.41–1.78	246	**1.24**	1.09–1.41	89	**0.95**	0.76–1.17	<*0.01*
Soft tissue sarcoma (171)	16	**3.10**	1.77–5.03	11	**2.11**	1.05–3.77	12	**2.04**	1.05–3.56	10	**2.62**	1.26–4.83	*0.56*
Melanoma of skin (172)	67	**1.53**	1.18–1.94	83	**2.25**	1.79–2.79	67	**1.92**	1.49–2.44	41	**2.17**	1.55–2.94	*0.11*
Other neoplasm of skin (173)	157	**3.05**	2.59–3.57	243	**3.42**	3.00–3.88	327	**3.47**	3.10–3.87	233	**3.06**	2.68–3.48	*0.93*
Bladder (188, 189.3, 189.4)	84	**2.34**	1.87–2.90	85	**1.37**	1.10–1.70	102	**1.29**	1.05–1.56	72	**1.40**	1.09–1.76	<*0.01*
Kidney (189) (−189.3, 189.4)	55	**2.10**	1.58–2.73	67	**1.86**	1.44–2.36	78	**1.98**	1.57–2.48	39	**1.82**	1.29–2.48	*0.62*
Thyroid gland (193)	22	**2.45**	1.54–3.71	12	**1.94**	1.00–3.38	10	**1.65**	0.79–3.04	12	**3.39**	1.75–5.92	*0.75*
Hodgkin's disease (201)	41	**7.87**	5.65–10.7	13	**3.89**	2.07–6.66	11	**3.46**	1.73–6.18	4	**2.34**	0.64–6.00	<*0.01*
Lymphoid leukaemia (204)	45	**5.44**	3.97–7.28	51	**3.91**	2.91–5.15	21	**1.28**	0.79–1.95	14	**1.19**	0.65–2.00	<*0.01*
Myeloid leukaemia (205)	17	**2.52**	1.47–4.04	15	**1.78**	1.00–2.93	12	**1.09**	0.57–1.91	7	**0.94**	0.38–1.94	<*0.01*

NHL, non-Hodgkin's lymphoma.

**Table 4:** Standardised incidence ratios of selected cancer sites after NHL by calendar period at NHL

	**Before 1975**			**1975–1983**			**1984–1990**			**1991 or after**			
**Cancer sites (ICD 9th rev.)**	**Obs.**	**SIR**	**95% CI**	**Obs.**	**SIR**	**95% CI**	**Obs.**	**SIR**	**95% CI**	**Obs.**	**SIR**	**95% CI**	***P* for trend**
Lip (140)	7	**0.91**	0.37–1.88	17	**1.65**	0.96–2.65	25	**2.82**	1.82–4.17	14	**2.62**	1.43–4.40	< *0.01*
Tongue (141)	9	**3.49**	1.60–6.63	12	**2.42**	1.25–4.22	18	**3.58**	2.12–5.66	4	**1.16**	0.32–2.98	*0.22*
Oropharynx (146)	3	**1.91**	0.39–5.59	7	**2.12**	0.85–4.38	6	**1.70**	0.62–3.70	8	**3.30**	1.43–6.51	*0.42*
Stomach (151)	93	**1.53**	1.24–1.88	82	**1.25**	0.99–1.55	70	**1.28**	0.99–1.61	40	**1.27**	0.91–1.73	*0.26*
Small intestine (152)	6	**2.41**	0.88–5.25	13	**3.36**	1.79–5.75	10	**2.49**	1.19–4.58	4	**1.38**	0.38–3.53	*0.29*
Colon (153)	79	**1.19**	0.94–1.48	163	**1.37**	1.16–1.59	143	**1.20**	1.02–1.42	111	**1.38**	1.14–1.66	*0.61*
Liver (155) (−155.2)	13	**1.69**	0.90–2.89	23	**1.80**	1.14–2.70	21	**1.55**	0.96–2.36	10	**1.07**	0.52–1.98	*0.23*
Nose and nasal cavity (160)	6	**3.28**	1.20–7.13	6	**2.44**	0.90–5.31	7	**2.91**	1.17–6.00	1	**0.65**	0.02–3.60	*0.20*
Lung (162)	131	**1.40**	1.17–1.66	296	**1.56**	1.39–1.75	277	**1.53**	1.36–1.72	150	**1.32**	1.11–1.54	*0.51*
Soft tissue sarcoma (171)	4	**1.03**	0.28–2.65	15	**2.49**	1.40–4.11	17	**2.84**	1.65–4.55	13	**3.09**	1.64–5.28	*0.06*
Melanoma of skin (172)	41	**2.48**	1.78–3.37	66	**1.69**	1.31–2.15	90	**1.97**	1.59–2.42	61	**1.82**	1.39–2.33	*0.39*
Other neoplasm of skin (173)	150	**3.31**	2.80–3.88	261	**3.14**	2.77–3.54	331	**3.47**	3.10–3.86	218	**3.17**	2.76–3.62	*0.95*
Bladder (188,189.3,189.4)	74	**1.84**	1.45–2.31	104	**1.49**	1.22–1.81	96	**1.36**	1.10–1.66	69	**1.43**	1.11–1.81	*0.11*
Kidney (189) (−189.3,189.4)	39	**1.70**	1.21–2.33	63	**1.73**	1.33–2.22	61	**1.61**	1.23–2.07	76	**2.94**	2.32–3.68	< *0.01*
Thyroid gland (193)	13	**2.57**	1.37–4.40	18	**2.48**	1.47–3.92	15	**2.06**	1.15–3.39	10	**1.94**	0.93–3.57	*0.41*
Hodgkin's disease (201)	24	**6.73**	4.31–10.0	22	**5.28**	3.31–8.00	11	**3.10**	1.55–5.54	12	**5.56**	2.87–9.72	*0.20*
Lymphoid leukaemia (204)	27	**2.85**	1.88–4.14	45	**2.92**	2.13–3.91	39	**2.61**	1.86–3.57	20	**2.07**	1.26–3.20	*0.24*
Myeloid leukaemia (205)	3	**0.50**	0.10–1.46	20	**1.82**	1.11–2.81	15	**1.47**	0.83–2.43	13	**2.03**	1.08–3.48	*0.09*

NHL, non-Hodgkin's lymphoma.

**Table 5:** Standardised incidence ratios of NHL after other first primary cancers by follow-up period

	**Less than 1 year**			**1–4 years**			**5–9 years**			**At least 10 years**				**Overall**		
**Cancer sites (ICD 9th revision)**	**Obs.**	**SIR**	**95% CI**	**Obs.**	**SIR**	**95% CI**	**Obs.**	**SIR**	**95% CI**	**Obs.**	**SIR**	**95% CI**	***P* for trend^a^**	**Obs.**	**SIR**	**95% CI**
Lip (140)	8	**0.88**	0.38–1.74	43	**1.40**	1.01–1.88	31	**1.11**	0.75–1.58	44	**1.06**	0.77–1.43	*0.21*	126	**1.16**	0.96–1.38
Tongue (141)	7	**2.11**	0.85–4.34	5	**0.74**	0.24–1.72	10	**2.13**	1.02–3.91	6	**1.25**	0.46–2.72	*0.35*	28	**1.43**	0.95–2.06
Salivary gland (142)	3	**1.41**	0.29–4.13	10	**1.74**	0.83–3.20	9	**1.87**	0.85–3.55	13	**1.43**	0.76–2.45	*0.62*	35	**1.61**	1.12–2.24
Mouth (143–145)	16	**3.15**	1.80–5.11	20	**1.76**	1.08–2.72	12	**1.55**	0.80–2.70	14	**1.86**	1.02–3.12	*0.92*	62	**1.96**	1.50–2.51
Oropharynx (146)	7	**3.38**	1.36–6.96	14	**3.58**	1.95–6.01	2	**0.82**	0.10–2.95	6	**2.81**	1.03–6.12	*0.38*	29	**2.75**	1.84–3.94
Nasopharynx (147)	4	**3.07**	0.84–7.86	7	**2.50**	1.00–5.14	5	**2.64**	0.86–6.16	8	**3.37**	1.45–6.64	*0.57*	24	**2.87**	1.84–4.26
Hypopharynx (148)	0	**0.00**	0.00–2.23	1	**0.45**	0.01–2.51	1	**0.90**	0.02–5.01	1	**1.17**	0.03–6.54	*0.47*	3	**0.51**	0.11–1.50
Pharynx unspecified (149)	0	**0.00**	0.00–11.6	0	**0.00**	0.00–8.37	2	**8.41**	1.02–30.4	0	**0.00**	0.00–19.6	*0.61*	2	**1.69**	0.20–6.10
Oesophagus (150)	7	**0.85**	0.34–1.75	5	**0.82**	0.26–1.90	3	**1.04**	0.21–3.03	0	**0.00**	0.00–1.41	*0.26*	15	**0.75**	0.42–1.24
Stomach (151)	38	**1.16**	0.82–1.59	34	**0.80**	0.55–1.11	35	**1.27**	0.88–1.77	48	**1.46**	1.07–1.93	<*0.01*	155	**1.14**	0.97–1.34
Small intestine (152)	1	**0.47**	0.01–2.64	9	**1.94**	0.89–3.69	7	**2.27**	0.91–4.68	1	**0.29**	0.01–1.63	*0.08*	18	**1.36**	0.81–2.15
Colon (153)	95	**1.29**	1.05–1.58	180	**1.07**	0.92–1.24	124	**1.11**	0.92–1.32	135	**1.25**	1.05–1.48	*0.19*	534	**1.16**	1.06–1.26
Rectum (154)	40	**0.84**	0.60–1.14	108	**1.00**	0.82–1.20	60	**0.87**	0.66–1.12	76	**1.07**	0.84–1.34	*0.71*	284	**0.96**	0.85–1.08
Liver (155) (−1552)	5	**1.76**	0.57–4.10	0	**0.00**	0.00–1.97	0	**0.00**	0.00–4.97	0	**0.00**	0.00–4.30	*NA*	5	**0.79**	0.26–1.85
Gallbladder, bile ducts (156)	3	**0.61**	0.13–1.77	3	**0.63**	0.13–1.83	2	**0.80**	0.10–2.90	3	**1.22**	0.25–3.57	*0.42*	11	**0.75**	0.37–1.34
Pancreas (157)	11	**0.96**	0.48–1.72	6	**1.18**	0.43–2.56	3	**1.26**	0.26–3.68	3	**1.19**	0.25–3.47	*0.98*	23	**1.07**	0.68–1.61
Peritoneum (158)	0	**0.00**	0.00–5.50	2	**2.04**	0.25–7.35	3	**5.62**	1.16–16.4	0	**0.00**	0.00–6.56	*0.60*	5	**1.82**	0.59–4.24
Nose and nasal cavity (160)	4	**2.14**	0.58–5.47	7	**1.68**	0.68–3.47	6	**2.02**	0.74–4.39	6	**1.57**	0.58–3.41	*0.91*	23	**1.79**	1.14–2.69
Larynx (161)	11	**1.13**	0.57–2.03	35	**1.29**	0.90–1.80	25	**1.16**	0.75–1.71	20	**0.83**	0.51–1.28	*0.12*	91	**1.10**	0.89–1.35
Lung (162)	86	**1.17**	0.93–1.44	73	**1.08**	0.85–1.36	35	**1.02**	0.71–1.42	37	**1.20**	0.84–1.65	*0.70*	231	**1.12**	0.98–1.27
Bone (170)	1	**0.95**	0.02–5.29	6	**2.39**	0.88–5.21	2	**0.93**	0.11–3.35	5	**1.07**	0.35–2.50	*0.19*	14	**1.35**	0.74–2.26
Soft tissue sarcoma (171)	14	**3.43**	1.88–5.76	21	**2.05**	1.27–3.13	12	**1.48**	0.76–2.58	21	**1.45**	0.90–2.21	*0.27*	68	**1.84**	1.43–2.33
Melanoma of skin (172)	60	**2.09**	1.60–2.70	137	**1.58**	1.32–1.87	99	**1.40**	1.14–1.71	99	**1.08**	0.88–1.32	<*0.01*	395	**1.42**	1.29–1.57
Other neoplasm of skin (173)	203	**2.65**	2.30–3.04	479	**2.11**	1.92–2.30	265	**1.62**	1.43–1.82	243	**1.54**	1.35–1.74	<*0.01*	1190	**1.90**	1.79–2.01
Female breast (174)	96	**1.01**	0.82–1.23	266	**0.91**	0.80–1.03	271	**1.20**	1.06–1.35	397	**1.39**	1.26–1.54	<*0.01*	1030	**1.15**	1.08–1.22
Male breast (175)	1	**0.97**	0.02–5.42	3	**1.04**	0.21–3.03	3	**1.60**	0.33–4.68	4	**2.33**	0.64–5.97	*0.28*	11	**1.46**	0.73–2.62
Cervix uteri (180)	14	**1.31**	0.72–2.20	40	**1.28**	0.91–1.74	50	**1.52**	1.13–2.01	167	**1.46**	1.25–1.70	*0.55*	271	**1.43**	1.27–1.61
Placenta (181)	0	**0.00**	0.00–237	0	**0.00**	0.00–60.8	0	**0.00**	0.00–37.3	0	**0.00**	0.00–6.35	*NA*	0	**0.00**	0.00–4.88
Corpus uteri (182)	25	**1.23**	0.79–1.81	60	**0.93**	0.71–1.20	74	**1.19**	0.93–1.49	127	**1.13**	0.94–1.34	*0.29*	286	**1.10**	0.98–1.24
Ovary (183)	10	**0.72**	0.34–1.32	14	**0.50**	0.28–0.84	30	**1.44**	0.97–2.05	62	**1.55**	1.19–1.99	<*0.01*	116	**1.13**	0.93–1.36
Other female genital (179,184)	9	**1.97**	0.90–3.75	8	**0.73**	0.31–1.43	11	**1.25**	0.62–2.23	17	**1.25**	0.73–2.00	*0.23*	45	**1.18**	0.86–1.58
Prostate (185)	194	**1.30**	1.12–1.50	359	**0.98**	0.88–1.08	191	**1.11**	0.96–1.28	78	**1.02**	0.81–1.28	*0.37*	822	**1.08**	1.00–1.15
Testis (186)	12	**5.97**	3.09–10.4	10	**1.38**	0.66–2.53	11	**1.30**	0.65–2.33	36	**1.57**	1.10–2.17	*0.63*	69	**1.70**	1.32–2.15
Other male genital (187)	4	**2.13**	0.58–5.47	11	**2.15**	1.08–3.86	4	**0.98**	0.27–2.51	7	**1.29**	0.52–2.66	*0.26*	26	**1.58**	1.03–2.31
Bladder (188,189.3,189.4)	59	**1.15**	0.87–1.48	159	**1.17**	1.00–1.37	103	**1.05**	0.86–1.28	113	**1.22**	1.01–1.47	*0.84*	434	**1.15**	1.04–1.26
Kidney (189) (−189.3,189.4)	31	**1.51**	1.02–2.14	57	**1.21**	0.91–1.56	39	**1.19**	0.85–1.63	41	**1.22**	0.88–1.66	*0.96*	168	**1.25**	1.07–1.46
Eye (190)	4	**1.56**	0.43–4.01	12	**1.56**	0.80–2.72	5	**0.83**	0.27–1.94	14	**1.49**	0.81–2.49	*0.95*	35	**1.36**	0.95–1.89
Brain, nervous system (191–192)	7	**1.09**	0.44–2.25	15	**1.99**	1.11–3.28	14	**2.35**	1.28–3.94	14	**1.11**	0.60–1.86	*0.10*	50	**1.53**	1.14–2.02
Thyroid gland (193)	22	**4.78**	3.00–7.24	20	**1.37**	0.84–2.12	19	**1.34**	0.81–2.09	41	**1.51**	1.08–2.04	*0.70*	102	**1.68**	1.37–2.04
Other endocrine gland (194, 164.0)	3	**5.61**	1.16–16.4	6	**4.53**	1.66–9.86	2	**1.84**	0.22–6.65	2	**1.34**	0.16–4.83	*0.10*	13	**2.93**	1.56–5.00
Hodgkin's disease (201)	14	**5.29**	2.89–8.87	39	**5.64**	4.01–7.71	46	**7.50**	5.49–10.0	70	**6.29**	4.90–7.94	*0.70*	169	**6.30**	5.39–7.32
Multiple myeloma (203)	13	**1.09**	0.58–1.86	38	**1.69**	1.19–2.31	20	**2.51**	1.53–3.87	8	**1.96**	0.84–3.85	*0.36*	79	**1.70**	1.34–2.11
Lymphoid leukaemia (204)	17	**1.63**	0.95–2.61	79	**2.92**	2.31–3.64	65	**4.36**	3.37–5.56	33	**3.90**	2.69–5.48	*0.05*	194	**3.19**	2.76–3.67
Myeloid leukaemia (205)	7	**1.55**	0.62–3.19	8	**1.27**	0.55–2.50	4	**1.83**	0.50–4.69	3	**2.31**	0.48–6.76	*0.33*	22	**1.54**	0.96–2.33
Other leukaemia (206–208)	8	**2.71**	1.17–5.35	15	**3.05**	1.71–5.03	6	**1.87**	0.68–4.06	9	**2.89**	1.32–5.48	*0.79*	38	**2.68**	1.89–3.67

NHL, non-Hodgkin's lymphoma.

a Excluding the category ‘Less than 1 year’.

NA, not applicable.

**Table 6:** Summary of the major findings

	**Trend (*P*<0.05)**				
**Cancer sites (ICD 9th revision)**	**With time since NHL diagnosis**	**With age at NHL diagnosis**	**With calendar period at NHL diagnosis**	**Risk of NHL as a second primary cancer and trend with time since the first cancer (*P*<0.05)**	**Comments**
Lip (140)	—	—	↗	1.16 (0.96–1.38) No trend	Common exposure or mechanism
Tongue (141)	—	—	—	1.43 (0.95–2.06) No trend	Common exposure or mechanism
Oropharynx (146)	—	—	—	2.75 (1.84–3.94) No trend	Common exposure or mechanism
Stomach (151)	↗	↘	—	1.14 (0.97–1.34) ↗	Treatment effect or misclassification of extranodal NHL
Small intestine (152)	—	—	—	1.36 (0.81–2.15) No trend	Detection bias
Colon (153)	—	—	—	1.16 (1.06–1.26) No trend	Misclassification of extranodal NHL
Liver (155) (−155.2)	—	—	—	0.79 (0.26–1.85) No trend	Common exposure or mechanism
Nose and nasal cavity (160)	—	—	—	1.79 (1.14–2.69) No trend	Common exposure or mechanism
Lung (162)	↗	↘	—	1.12 (0.98–1.27) No trend	Treatment effect +common exposure
Soft tissue sarcoma (171)	—	—	—	1.84 (1.43–2.33) No trend	Common exposure or mechanism
Melanoma of skin (172)	↘	—	—	1.42 (1.29–1.57) ↘	Common exposure or mechanism
Other neoplasm of skin (173)	—	—	—	1.90 (1.79–2.01) ↘	Common exposure or mechanism
Bladder (188, 189.3, 189.4)	↗	↘	—	1.15 (1.04–1.26) No trend	Treatment effect +common exposure
Kidney (189) (−189.3, 189.4)	—	—	↗	1.25 (1.07–1.46) No trend	Detection bias
Thyroid gland (193)	—	—	—	1.68 (1.37–2.04) No trend	Common exposure or mechanism
Hodgkin's disease (201)	↗	↘	—	6.30 (5.39–7.32) No trend	Treatment, common exposure or misclassifciation
Lymphoid leukaemia (204)	—	↘	—	3.19 (2.76–3.67) No trend	Misclassification and common exposure
Myeloid leukaemia (205)	—	↘	—	1.54 (0.96–2.33) No trend	Treatment effect

NHL, non-Hodgkin's lymphoma.
